# Functional Genomics and Disease in 2003

**DOI:** 10.1002/cfg.329

**Published:** 2003-10

**Authors:** Michael J. Taussig

**Affiliations:** ESF Programme in Integrated Approaches to Functional Genomics The Babraham Institute Cambridge CB2 4AT UK


**Comparative and Functional Genomics**
*Comp Funct Genom* 2003; **4**: 516–517.
Published online in Wiley InterScience (www.interscience.wiley.com). **DOI:** 10.1002/cfg.329

**Conference Editorial**

### Functional genomics and disease in 2003

Michael J. Taussig*
*Chairman, ESF Programme in Integrated Approaches to Functional Genomics, The Babraham Institute, Cambridge CB2 4AT, UK*

**Correspondence to: Michael J. Taussig, ESF Programme in Integrated Approaches to Functional Genomics, The Babraham Institute, Cambridge CB2 4AT, UK. E-mail: mike.taussig@bbsrc.ac.uk*

Received: 1 August 2003
Revised: 4 August 2003
Accepted: 5 August 2003

**Keywords:** functional genomics; proteomics; arrays; disease; diagnosis; prognosis; sequence variation; ethics

2003 is a highly celebratory year for genomics, with the 50th anniversary of the publication of the double helical structure of DNA (April 1953) and the announcement of the completion of the human genome sequence (April 2003). The structure of DNA, arguably the most important biological advance of the 20th century, and the human genome project have fundamentally affected our view of biology. The determination of the DNA structure, in a union of physics, chemistry and biology, revealed how information is encoded by genes and how those genes are replicated; the completion of the human genome sequence offers the prospect of disease prediction, prevention and cure, as well as a deeper understanding of our own evolution.

Having determined the informational content of many genomes as specified in the DNA, we are now moving on to elucidating how gene products function and how the chemistry of life is translated into complex cellular organization. This is the area covered by functional genomics, the exploration of gene function on a global scale. Functional genomics makes use of revolutionary technological advances, characteristically capable of very high sample throughput and producing vast amounts of data, which require computational processing for interpretation. Array technologies have proved particularly powerful. DNA arrays can reveal genetic variation or monitor expression of integrated sets or all of the genes of an organism at the mRNA level in one experiment, with small samples of tissue; the more recent antibody arrays can similarly reveal the protein levels, while protein or whole-proteome chips give the opportunity for parallel functional analysis. The 'classical' proteomics approaches of high resolution two-dimensional gel electrophoresis or liquid chromatography, combined with mass spectrometry, are capable of separating and identifying thousands of proteins, revealing changes in protein expression and the composition of intracellular protein complexes. Protein–protein interactions can be analysed *in vivo* by the yeast two-hybrid approach and its derivatives. Antibodies, the most familiar form of binding molecules, are again proving their immense value in analysing proteins of unknown function identified from the genome sequence. Elegant knockdown strategies, such as RNAi or mutagenesis approaches, can specifically silence or alter individual genes, the effects of which can be observed on a global scale in genetically amenable 'model organisms' such as yeast,

Copyright © 2003 John Wiley & Sons, Ltd.

nematode, fruit fly and mouse, leading to new phenotypes from which gene function may be deduced. Bioinformatics is a vital growth area without which the data cannot be made accessible, organized and understood. The application of these technologies to the understanding and treatment of human disease was the focus of this meeting.

What can we expect the benefits of functional genomics research to be for human health? Taking cancer as one example, functional genomics technologies, as exquisitely sensitive high-throughput tools, allow for improved characterization of the varieties of types and subtypes of tumours. Transcriptional and protein profiling reveal an enormous number of genes that change activity together as part of an interconnected network. One result of observing the clusters of genes which change in expression has been in improving classification of cancers, with major clinical significance in assisting earlier diagnosis, prognosis or therapy. As regards drug development, the expectation is that the human genome information and functional genomics research will lead to cures through the identification of altered molecular pathways, the selection of new tumour-specific targets and the development of drugs against each tumour type. The success of the anti-CML drug Gleevec™ (and others in the wake of this) suggests that the 'designer drugs' approach will deliver. However, given the number of cancer varieties, the huge cost of drug development and many years of clinical testing and validation, this cannot typically be expected to occur very quickly. A realistic short-term goal for functional genomics, which will increase survival, is improved early diagnosis, currently lacking for many cancers, through the discovery of protein markers in body fluids, particularly serum. In particular, proteomics technologies of mass spectrometry and antibody arrays offer the possibility of screening for early disease. Improved diagnostic methods for early disease detection are probably where the promise of functional genomics will be realized most rapidly.

A major potential benefit of genome information is prevention of disease by identifying susceptible individuals through their genomic profile, correlating DNA sequence variation (SNPs, haplotypes) with disease susceptibility. While the power of prediction for single gene disorders conferred by the sequence is very good, it is currently much more modest for the common, multifactorial diseases, for which the phenotypes are likely to be caused by combinations of alleles. Functional genomics approaches may allow us to understand how the interactions among gene products ultimately give rise to phenotypes of complex diseases. However, this will be very challenging and many components will have to be integrated to understand how the disease phenotypes are generated.

Genomic research and its implications have raised a range of issues for ethical and legal consideration. Health predictions and early detection are clearly of value where treatment or lifestyle modification are effective, but where there is little or nothing to be done the information may well produce major stress for the individual and may adversely impact life insurance or employment. What should be revealed and how the information is obtained and used are becoming important considerations. Population-based collections of tissues and medical histories are invaluable resources for genomics but raise questions of the rights of the individual and of the researchers, as well as the interests of companies keen to utilize the information in drug development. On the commercial front, there are valid concerns over patenting of genome information and its commercial exploitation. To be of benefit to society, the results of genomics research require commercial development by biotech and pharmaceutical companies, and it is clearly in the financial interests of universities and institutions to facilitate technology transfer. The problem is how to reconcile the academic and industrial ethos — sharing of results *versus* confidentiality and patent rights. The results of publicly funded research should be made freely available for the good of society, as has happened with the human genome project, but the commercial sector cannot operate in quite the same way. While academic researchers should not be restricted in what they can do and publish, at the same time there has to be an easy transition into patenting, commercial application and development where different rules apply. Problems such as these will have to be addressed to achieve the widely anticipated benefits of the understanding of the human genome in this 50th anniversary year of DNA.

### Acknowledgements

I am grateful to Gert-Jan van Ommen and Ulf Landegren for comments on this article.

Copyright © 2003 John Wiley & Sons, Ltd.

